# A Joint Power and Channel Scheduling Scheme for Underlay D2D Communications in the Cellular Network

**DOI:** 10.3390/s19214799

**Published:** 2019-11-04

**Authors:** Zefang Lin, Hui Song, Daru Pan

**Affiliations:** School of Physics and Telecommunication Engineering, South China Normal University, Guangzhou 510631, China; 2017021649@m.scnu.edu.cn

**Keywords:** D2D communications, energy efficiency, power control, resource blocks allocation, cellular network

## Abstract

Device-to-device (D2D) communication, as one of the promising candidates for the fifth generation mobile network, can afford effective service of new mobile applications and business models. In this paper, we study the resource management strategies for D2D communication underlying the cellular networks. To cater for green communications, our design goal is to the maximize ergodic energy efficiency (EE) of all D2D links taking into account the fact that it may be tricky for the base station (BS) to receive all the real-time channel state information (CSI) while guaranteeing the stability and the power requirements for D2D links. We formulate the optimization problem which is difficult to resolve directly because of its non-convex nature. Then a novel maximum weighted ergodic energy efficiency (MWEEE) algorithm is proposed to solve the formulated optimization problem which consists of two sub-problems: the power control (PC) sub-problem which can be solved by employing convex optimization theory for both cellular user equipment (CUE) and D2D user equipment (DUE) and the channel allocation (CA) sub-problem which can be solved by obtaining the weighted allocation matrix. In particular, we shed light into the impact on EE metric of D2D communication by revealing the nonlinear power relationship between CUE and DUE and taking the QoS of CUEs into account. Furthermore, simulation results show that our proposed algorithm is superior to the existing algorithms.

## 1. Introduction

With the explosive growth of mobile handheld devices which has led to an increasing demand for higher data rates and radio spectrum resources in the past decade, a wireless access world with lower power consumption, lower latency and higher data rates is anticipated. By allowing two adjacent user equipments (UEs) to communicate directly rather than through the BS or core network [1,2,3,4], D2D communications, as a key technology for the fifth generation (5G), can bring some benefits such as higher link stability, lower power consumption, improving the edge user communication efficiency and significantly improving spectrum efficiency (SE) which can be achieved by DUEs sharing the licensed spectrum channels with CUEs [5,6]. From the perspective of the BS or core network, D2D communication can greatly reduce their communication data stream load, which is an urgent problem to be addressed due to the increasing density of mobile networks and the high cost of base station construction. Moreover, D2D communication mode can be chosen to overlay D2D communication in the cellular networks if dedicated bandwidth is reserved for DUEs. Although D2D communication technology is provided with promising potential benefits, there come with it a few technical challenges such as stable establishment of communication links, effective device discovery scheme, implementing communication mode selection and coordinating the interference between primary user equipments and secondary user equipments, i.e., CUEs and DUEs respectively. Selection mode has been considered in [7,8,9]. In this paper, we focus on the interference management for underlay D2D communication in the cellular network.

## 2. Related Works

In recent years, various resource management technique-based literature has attempted to conquer the challenges that D2D technology is confronted with in order to take full advantage of the benefits of D2D communication underlying the cellular networks. The following papers [10,11,12,13,14,15,16,17,18,19,20,21,22,23,24,25,26,27,28,29,30,31,32,33] represent some of the research aiming to execute interference management. The authors of [10,11,12] investigated the resource allocation mechanism under a cellular system from the game theory point of view. The matching theory and the idea of cheating was introduced to solve the resource allocation problem that was modeled as a stable marriage problem to find an optimal matching with maximal throughput objective [13]. The authors of [14] consider a multi-cell scenario where the receivers of D2D pairs were assumed as victim nodes and CUEs as aggressor nodes. The authors aimed to improve the SINR of DUEs by minimizing inter-cell interference at the cell edge area. In [15], the authors preformed the radio spectrum resource allocation for D2D communication underlaying the network where an DUE pair is allowed to reuse the uplink resource by using graph theory. Moreover, for the purpose of aiming to maximize the ergodic sum rate, the feasible power region of CUE and DUE was described according to the outage probability requirement. A power allocation scheme with double sum-rate maximization optimal problem was presented in [16]. The study in [17] demonstrated a channel assignment algorithm including optimal channel assignment algorithm based on dynamic programming and a cluster-based sub-optimal channel assignment algorithm while considering the partial CSI. The concept of proportional fairness was mentioned in [18] and the authors proposed a resource scheduling mechanism which considered the time-varying feature of channel condition to maximize the network throughput and users’ fairness in the underlay D2D communication. The authors of [19] proposed an overlapping coalitional game where each DUE can reuse multiple resource blocks (RBs) and multiple DUEs can share single spectral with each other. Moreover, the proposed scheme ensured the system-wide security and improved the performance by maximizing the system sum rate. In [20], the authors study a resource allocation problem to maximize the overall network throughput and proposed a three-step scheme which includes admission control, power allocation and bipartite matching. In the power allocation stage, it searches for the optimal power solution of CUEs and DUEs in the region where the power value of CUE and DUE range from zero to the maximum power value. The authors of [21] performed the resource management mechanism based only on slowly varying large-scale fading information of wireless channels with the goal of maximizing the ergodic capacity of CUEs when sharing a spectrum with DUEs. In particular, the power allocation problem was solved by using the bisection search method according to the power feasible regions that depend on magnitudes of the maximum power of CUE and DUE. The aforementioned literature mainly chooses the throughput or SE as optimization objective function, which do not consider the case where the increased SE may lead to the deterioration of EE of the system [22,23]. The authors in [24] investigated an energy-efficient downlink resource reuse scheme for underlay D2D communication and proposed an iterative algorithm. The power relationship was described first. Then optimization problem was solved by KKT condition. In [25], the authors committed to finding Pareto optimal solutions for the resource allocation problem which was formulated as an uncertain multi-objective optimization problem to maximize EE and SE of CUEs. In [26], the authors modeled the resource management problem as a MINLP problem for green communication and split it into two sub-problems. Then the spectrum allocation sub-problem was solved by introducing a heuristic algorithm and the power solution was obtained by dealing with the convex optimization problem. A joint resource management scheme considering three different modes was proposed in [27] and the authors formulated the optimization problem as a three-dimensional problem to maximize the the overall EE of D2D communications. In [28], considering both the network EE and the max-min individual EE, matching theory was introduced to solve the channel allocation problem and then a transmission power solution was obtained via fractional programming. The authors in [29] study the joint energy harvesting time slot allocation, power and resource block allocation problem while guaranteeing the QoS of CUEs and the energy harvesting constraints of D2D links. In addition, the iterative algorithm based on Dinkelbach and Lagrangian constrained optimization was proposed to tackle the original nonconvex problem. The authors of [30] investigated the downlink resource allocation problem which was formulated to maximize the weighted EE and solved by using game-theoretic learning approach in the energy harvesting-based D2D communication network. In particular, the authors decomposed the original optimization problem into two subproblems and modeled them as two exact potential games. Most of the resource allocation schemes ignored the channel uncertainty and thus assumed that BS can acquire the CSI of all communication links [31,32,33]. However, it is worth noting that this is unrealistic, especially for high-density network structures. In addition, the small-scale channel fading or statistical CSI is easily overlooked and then the proposed resource management schemes cannot highlight the actual performance of the system [15]. Thus, the important practical significance of CSI of communication links in underlay D2D cellular networks will be revealed in this study.

In this paper, we study the power control and channel allocation problem for underlay D2D communication over the uplink that joint large-scale channel and statistical small-scale channel. We consider the mobility of nodes and the fact that BS cannot acquire the real-time CSI of all communication links. Furthermore, our design objective is to maximize the weighted ergodic EE of D2D communications with the fact that the limited battery capacity of mobile device fails the long-lasting communication. In addition, we allow one D2D pair to reuse at least one orthogonal RB that is allocated to CUE. Thus, D2D communications can further utilize the spectrum while reducing the interference experienced by the CUE. The contributions of this paper are summarized as follows.

- We perform a two-step resource management problem on the joint small-scale and large-scale channel and take the statistical SCI into account; thus, the optimization problem aiming to maximize the ergodic EE of D2D communications is formulated. Moreover, we incorporate the outage probability constraint into the problem.
- We analyze the outage probability constraint and explore the relationship of transmit power between DUE and CUE to simplify the resulting non-convex optimization problem. To the best of our knowledge, for the existing literatures, this is the first work that introduces the nonlinear relationship of transmit power between CUE and DUE, which is more robust than the functional relationship other researches involved from the perspective of linear relationship.
- We transform the MINLP problem into two sub-problems, i.e., the PC sub-problem for both CUEs and CUEs and CA sub-problem. Besides, we allow DUEs to asynchronously reuse the RBs of CUEs so that each DUE only suffers from the interference from one CUE. In the spectrum allocation phase, we introduce weight coefficients between ergodic EE of DUEs and received interference from the transmitters of DUEs at BS to maintain trade-off between maximizing energy efficiency and minimizing interference.
- Three algorithms are used for comparison and the simulation results show the superiority of the proposed algorithm in terms of EE and the received interference at BS.

The remainder of this paper is organized as follows. Section 3 presents the system model and the ergodic EE-maximum optimization problem formulation. We solve the formulated problem by separating it into two sub-problems and propose the maximizing weighted ergodic EE (MWEEE) algorithm in Section 4. In Section 5, we analyze the simulation results and evaluate the performance of our proposed algorithm. Finally, Section 6 concludes the paper.

## 3. System Model and Problem Formulation

### 3.1. System Model

**Figure 1:**
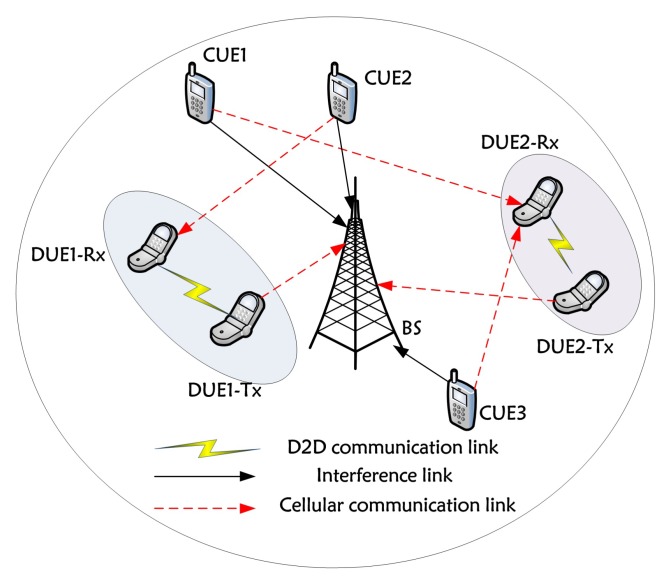
System scenario for device-to-device (D2D) communications underlaying cellular network.

As shown in Figure 1, we consider a single cell D2D communication underlaying two-layer cellular networks, where DUEs and CUEs are randomly distributed in the cell with one BS in the center. In the network, we assume there are *M* pairs of adjacent DUEs which reuse *K* orthogonal uplink RBs that was pre-allocated to *K* CUEs. We represent the set of CUEs as C=1,2,3,…,K and represent the set of D2D pairs as D=1,2,3,…,M.

Let $h_{m,k}$ denote the channel gain between the transmitter of *m*-th DUE pair transmitting on *k*-th resource block and the BS. Note that in this paper we jointly consider large-scale fading and small-scale fading and exploit the statistical CSI instead of instantaneous CSI. The channel gain $h_{m,k}$ can be expressed as follows:

(1) $$h_{m,k}=g_{m,k}\cdot K_{m,k}=g_{m,k}\cdot \Lambda_{m,k}\cdot L_{p}\cdot d^{-\gamma },$$

where $g_{m,k}$ is the statistical channel gain, i.e., the small-scale fast fading part, and is considered to be exponential distribution with unit mean, $\Lambda_{m,k}$ is log-normal shadow fading random variable with a standard deviation ζ, $L_{p}$ is the pathloss constant, *d* is the distance between *m*-th user and *k*-th user, γ is the decay exponent and $K_{m,k}$ is the large-scale channel fading component. Similarly, we can define $h_{m}$, $h_{k,B}$, $h_{k,m}$ as the channel gain between the *m*-th DUE pair, between the *k*-th CUE and BS, and between *k*-th CUE and receiver of *m*-th DUE pair, respectively.

Then the signal-to-interference-plus-noise (SINR) for the *k*-th CUE at BS and *m*-th D2D pair can be respectively given by

(2) $$SIN{R_{k}}^{c}=\frac{{P_{k}}^{c}h_{k,B}}{N_{0}+{P_{m,k}}^{d}h_{m,k}}$$

and

(3) $$SIN{R_{m,k}}^{d}=\frac{{P_{m,k}}^{d}h_{m}}{N_{0}+{P_{k}}^{c}h_{k,m}},$$

where ${P_{m,k}}^{d}$ and ${P_{k}}^{c}$ indicates the transmit power of *m*-th DUE on channel *k* and *k*-th CUE respectively, $N_{0}$ is the additive white Gaussian noise variance with zero mean.

### 3.2. Problem Formulation

As we aim to maximize the ergodic EE of all the DUEs based the large-scale fading CSI and statistical fast-scale fading CSI, first the EE of D2D communications can be denoted as $U_{ee}$, which is defined as follows:

(4) $$U_{ee}=\frac{\sum_{m=1}^{M}\sum_{k=1}^{K}\lambda_{m,k}R_{m,k}}{\sum_{m=1}^{M}\sum_{k=1}^{K}\lambda_{m,k}{P_{m,k}}^{d}+\sum_{m=1}^{M}P_{cir}},$$

where $R_{m,k}=log_{2}\left(1+SIN{R_{m,k}}^{d}\right)$ is the data rate of *m*-th pair of DUE on *k*-th channel, $P_{cir}$ is the circuit power of the considered DUE pair and assumes as a constant value. Then the ergodic EE of D2D communication is given as $C_{ee}=E\left[U_{ee}\right]$. Note that the expectation of E[.] is taken over the small-scale fast fading distribution. To this point, we can formulate the ergodic EE optimization problem as follows:

(5) $$\begin{array}{cc} P1:\;\;\;\;\;\;\;\;\;\;\;\;\;\; & \max_{\lambda_{m,k},\;{P_{m,k}}^{d},\;{P_{k}}^{c}}E[\frac{\sum_{m=1}^{M}\sum_{k=1}^{K}\lambda_{m,k}R_{m,k}}{\sum_{m=1}^{M}\sum_{k=1}^{K}\lambda_{m,k}{P_{m,k}}^{d}+\sum_{m=1}^{M}P_{cir}}]\\ s.t.\;\;\;\; & C1:\mathrm{Pr}\left\{{SINR_{m,k}}^{d}\leq SINR_{0}\right\}\leq p_{0},\;\forall m\in D,k\in C\\ \; & C2:\sum_{m=1}^{M}\lambda_{m,k}\leq 1,\;\forall k\in C\\ \; & C3:\sum_{k=1}^{K}\lambda_{m,k}{P_{m,k}}^{d}\leq {P_{d}}^{\max},\;\forall m\in D \end{array}$$

where Pr {.} denotes the probability of input, $SINR_{0}$ is the minimum SINR requirement DUEs must meet, $p_{0}$ is the tolerable outage probability for DUEs to keep transmission and ${P_{d}}^{\max}$ is the maximum transmitting power of DUE. C1 ensures transmission reliability requirement of DUE on each channel. Note that we base the resource management scheme on statistical CSI instead of instantaneous CSI and guarantee the reliability of D2D link through controlling the probability of outage events. This event is probabilistically distributed, depending on the signal-to-noise ratio of the link and its channel fading distribution model. Thus C1 here is probability and not the actual inequality. C2 represents the situation that each resource block cannot be reused by more than one DUE. C3 means that each DUE pair cannot exceed its maximum transmit power. In order to make the optimization problem tractable, we transform it into an equivalent form. From C1, the following Lemma 1 is obtained.

**Lemma** **1.** *In order to achieve the optimal EE of D2D communications while guaranteeing the QoS of CUE and the reliable link of m-th DUE on k-th channel, i.e., the minimum effective communication capability, the transmission power of k-th CUE should be set as:*

(6) $${P_{k}}^{c}=\frac{K_{m}{P_{m,k}}^{d}}{K_{k,m}SINR_{0}}\left(\frac{e^{-\frac{N_{0}SINR_{0}}{K_{m}{P_{m,k}}^{d}}}}{1-p_{0}}-1\right)$$

**Proof** **of** **Lemma** **1.** See Appendix A.

To guarantee ${P_{k}}^{c}\geq 0$, The following condition must be satisfied, i.e.,

(7) $${P_{m,k}}^{d}\geq \frac{-SINR_{0}N_{0}}{K_{m}\log\left(1-p_{0}\right)}$$

P1 is a nonlinear fractional programming problem due to the fractional objective. To make P1 easier to solve, after substituting the ${P_{k}}^{c}$ in formula (6) into P1, we let ${C_{ee}}^{*}$ denote the maximum ergodic EE of D2D communications and it can be written as follows:

(8) $${C_{ee}}^{*}=E\left[\frac{\sum_{m=1}^{M}\sum_{k=1}^{K}{\lambda^{*}}_{m,k}\log2\left(1+\frac{P^{*}{{}_{m,k}}^{d}h_{m}}{A_{1}A_{3}P^{*}{{}_{m,k}}^{d}e^{\frac{-A_{2}}{P^{*}{{}_{m,k}}^{d}}}-A_{1}P^{*}{{}_{m,k}}^{d}+N_{0}}\right)}{\sum_{m=1}^{M}\sum_{k=1}^{K}{\lambda^{*}}_{m,k}P^{*}{{}_{m,k}}^{d}+\sum_{m=1}^{M}P_{cir}}\right]$$

where $A_{1}=\frac{K_{m}h_{k,m}}{SINR_{0}K_{k,m}}$, $A_{2}=\frac{SINR_{0}N_{0}}{K_{m}}$, $A_{3}=\frac{1}{1-p_{0}}$, ${\lambda^{*}}_{m,k}$ and $P^{*}{{}_{m,k}}^{d}$ are assumed as the optimal solutions. Then, the Dinkelbach method [34,35,36] is applied to transform the fractional objective function into a corresponding subtractive form according to the following Proposition 1.

**Proposition** **1.** *Solving the optimization problem is equivalent to the problem given by*$\varphi ({C_{ee}}^{*})=0$ and the function of $\varphi ({C_{ee}}^{*})$ *is defined as follows:*

(9) $$\begin{array}{c} \varphi \left({C_{ee}}^{*}\right)=E\left[\sum_{m=1}^{M}\sum_{k=1}^{K}{\lambda^{*}}_{m,k}\log_{2}\left(1+\frac{P^{*}{{}_{m,k}}^{d}h_{m}}{A_{1}A_{3}{P_{m,k}}^{d}e^{\frac{-A_{2}}{P^{*}{{}_{m,k}}^{d}}}-A_{1}P^{*}{{}_{m,k}}^{d}+N_{0}}\right)\right]-C_{ee}(\sum_{m=1}^{M}\sum_{k=1}^{K}{\lambda^{*}}_{m,k}P^{*}{{}_{m,k}}^{d}+\sum_{m=1}^{M}P_{cir}) \end{array}$$

Note that the E[.] is taken over the $g_{k,m}$ and $g_{m}$, thus only the first item is added with E[.]. Since the proof of Proposition 1 is similar to the proof in [34] and the convergence analysis of the problem can also be found in the section 2 in [34], we omit the proof here. Then the optimization problem can be described as follows:

(10) $$\begin{array}{cc} P2:\;\;\;\;\;\\ \; & \begin{array}{c} \max_{\lambda_{m,k},\;{P_{m,k}}^{d}}E\left[\sum_{m=1}^{M}\sum_{k=1}^{K}\lambda_{m,k}\log_{2}\left(1+\frac{{P_{m,k}}^{d}h_{m}}{A_{1}A_{3}{P_{m,k}}^{d}e^{\frac{-A_{2}}{{P_{m,k}}^{d}}}-A_{1}{P_{m,k}}^{d}+N_{0}}\right)\right]-C_{ee}(\sum_{m=1}^{M}\sum_{k=1}^{K}\lambda_{m,k}{P_{m,k}}^{d}+\sum_{m=1}^{M}P_{cir}) \end{array}\\ s.t.\;\;\;\; & C1:\sum_{m=1}^{M}\lambda_{m,k}\leq 1,\;\forall k\in C\\ \; & C2:\sum_{k=1}^{K}\lambda_{m,k}{P_{m,k}}^{d}\leq {P_{d}}^{\max},\;\forall m\in D\\ \; & C3:{P_{m,k}}^{d}\geq \frac{-SINR_{0}N_{0}}{K_{m}\log\left(1-p_{0}\right)},\;\forall m\in D,\;k\in C \end{array}$$

□

## 4. Proposed Power Control and Channel Allocation Scheme

It is observed that the P2 is an MINLP problem which is generally difficult to deal with due to the existence of integer variable $\lambda_{m,k}$ and the non-convex of objective function. Therefore, we commit to a novel and effective approach that the problem is decomposed into two sub-problems. Then we propose low complexity algorithms for each sub-problem.

### 4.1. Power Control

In this part, we solve the problem given the $\lambda_{m,k}=1$, so the P2 is performed with respect to $P_{m,k}$. Note that here we consider the asynchronous channel reuse. This means that only one DUE update its power independently at each time and thus the condition of $\sum_{m=1}^{M}\lambda_{m,k}\leq 1$ is satisfied for P2. Besides, reviewing the model scenario where each block resource that pre-allocated to CUE can only be reused by one DUE and each DUE can reuse more than one block, and the definition for SINR of DUE, the interference received by DUE only comes from CUE and Gaussian white noise, no other DUEs interference, which satisfy the C1 of P2. Considering the optimization variable is independent of $g_{k,m}$ and $g_{m}$ over which E[.] is taken, we take the $U_{ee}$ instead of $C_{ee}$ for simplicity. Then the EE optimization problem is expressed as follows:

(11) $$\begin{array}{cc} P3:\;\;\;\;\;\;\\ \; & \max_{{P_{m,k}}^{d}}\sum_{m=1}^{M}\sum_{k=1}^{K}\log_{2}\left(1+\frac{{P_{m,k}}^{d}h_{m}}{A_{1}A_{3}{P_{m,k}}^{d}e^{\frac{-A_{2}}{{P_{m,k}}^{d}}}-A_{1}{P_{m,k}}^{d}+N_{0}}\right)-U_{ee}(\sum_{m=1}^{M}\sum_{k=1}^{K}{P_{m,k}}^{d}+\sum_{m=1}^{M}P_{cir})\\ s.t.\;\;\;\; & C1:\sum_{k=1}^{K}{P_{m,k}}^{d}\leq {P_{d}}^{\max},\;\forall m\in D\\ \; & C2:{P_{m,k}}^{d}\geq \frac{-SINR_{0}N_{0}}{K_{m}\log\left(1-p_{0}\right)},\;\forall m\in D,k\in C \end{array}$$

We denote

(12) $$R\left({P_{m,k}}^{d}\right)=\log_{2}\left(1+\frac{{P_{m,k}}^{d}h_{m}}{A_{1}A_{3}{P_{m,k}}^{d}e^{\frac{-A_{2}}{{P_{m,k}}^{d}}}-A_{1}{P_{m,k}}^{d}+N_{0}}\right)$$

Unfortunately, the P3 is a non-convex optimization problem and is hard to solve due to the $R({P_{m,k}}^{d})$ which is in general non-concave. To solve this thorny situation, we first substitute an item of the exponential function form in the $R({P_{m,k}}^{d})$, i.e., $e^{\frac{-A_{2}}{{P_{m,k}}^{d}}}$, with its first-order Taylor expansion as follows:

(13) $$e^{-\frac{A_{2}}{{P_{m,k}}^{d}}}\approx e^{-\frac{A_{2}}{{P_{m,k}}^{d}\left(0\right)\;\;}}+\frac{A_{2}}{{\left({P_{m,k}}^{d}\left(0\right)\;\right)}^{2}}e^{-\frac{A_{2}}{{P_{m,k}}^{d}\left(0\right)\;}}\left({P_{m,k}}^{d}-{P_{m,k}}^{d}\left(0\right)\;\right)$$

where ${P_{m,k}}^{d}\left(0\right)\;$ is the initial power of *m*-th DUE on *k*-th channel and can be updated by ${P_{m,k}}^{d}\left(0\right)\;={P_{m,k}}^{d}\left[j-1\right]$, where *j* is the number of iteration.

Replacing the formula (13) into $R({P_{m,k}}^{d})$, we can get the $\tilde{R}\left({P_{m,k}}^{d};{P_{m,k}}^{d}\left(0\right)\right)$ as follows:

(14) $$\tilde{R}\left({P_{m,k}}^{d};{P_{m,k}}^{d}\left(0\right)\right)=\log_{2}\left(1+\frac{{P_{m,k}}^{d}h_{m}}{B_{1}{\left({P_{m,k}}^{d}\right)}^{2}+B_{2}{P_{m,k}}^{d}+N_{0}}\right)$$

where $B_{1}=\frac{A_{1}A_{2}A_{3}}{{\left({P_{m,k}}^{d}\left(0\right)\;\right)}^{2}}e^{\frac{-A_{{}_{2}}}{{P_{m,k}}^{d}\left(0\right)}}$, $B_{2}=\left(A_{1}A_{3}-\frac{A_{1}A_{2}A_{3}}{{\left({P_{m,k}}^{d}\left(0\right)\;\right)}^{2}}\right)e^{\frac{-A_{{}_{2}}}{{P_{m,k}}^{d}\left(0\right)}}-A_{1}$. Note that $R({P_{m,k}}^{d})$ is approximated around the initial value ${P_{m,k}}^{d}\left(0\right)$ by the function $\tilde{R}\left({P_{m,k}}^{d};{P_{m,k}}^{d}\left(0\right)\right)$. Reviewing the Lemma 1 and the definition of $SIN{R_{m,k}}^{d}$, from $\tilde{R}\left({P_{m,k}}^{d};{P_{m,k}}^{d}\left(0\right)\right)$ we can intuitively consider ${P_{k}}^{c}$ is the quadratic function of ${P_{m,k}}^{d}$, which describes the real power collaboration between DUEs and CUEs.

Then, for the purpose of converting $\tilde{R}\left({P_{m,k}}^{d};{P_{m,k}}^{d}\left(0\right)\right)$ into a concave function, we exploit a lower bound for ln(1+Z) given by Lemma 2 [37] as follows:

**Lemma** **2.** *For any given* Z≥0 *and* $\tilde{Z}\geq 0$ *, we have* ln(1+Z)≥αln(Z)+β *, where α and β are the approximation coefficients which can be updated given the newly obtained* $\tilde{Z}$ *and are determined as follows:*

(15) $$\begin{array}{c} \alpha =\frac{\tilde{Z}}{1+\tilde{Z}},\\ \beta =\ln\left(1+\tilde{Z}\right)-\alpha \ln\left(\tilde{Z}\right). \end{array}$$

*The lower bound is tight at* $Z=\tilde{Z}$ *.*

Note that in $\tilde{R}\left({P_{m,k}}^{d};{P_{m,k}}^{d}\left(0\right)\right)$, $B_{1}\geq 0$ is established. To guarantee $Z=\frac{{P_{m,k}}^{d}h_{m}}{B_{1}{\left({P_{m,k}}^{d}\right)}^{2}+B_{2}{P_{m,k}}^{d}+N_{0}}\geq 0$, the condition of $B_{2}\geq 0$ must be satisfied, i.e.,

(16) $$e^{\frac{A_{{}_{2}}}{{P_{m,k}}^{d}\left(0\right)}}+A_{2}A_{3}{\left({P_{m,k}}^{d}\left(0\right)\;\right)}^{2}\leq A_{3}$$

It means that ${P_{m,k}}^{d}\left(0\right)$ and the optimization variable ${P_{m,k}}^{d}$ must satisfy the formula (16). Thus we define

(17) $$\Omega =\{{P_{m,k}}^{d}\;|e^{\frac{A_{{}_{2}}}{{P_{m,k}}^{d}}}+A_{2}A_{3}{\left({P_{m,k}}^{d}\;\right)}^{-2}\leq A_{3}\}$$

Apply the Lemma 2 to the $\tilde{R}\left({P_{m,k}}^{d};{P_{m,k}}^{d}\left(0\right)\right)$ and let ${P_{m,k}}^{d}=e^{{\tilde{P}}_{m,k}^{d}}$. Then the relaxed $\tilde{R}\left({P_{m,k}}^{d};{P_{m,k}}^{d}\left(0\right)\right)$ can be obtained as follows:

(18) $$\tilde{R}\left({{\tilde{P}}_{m,k}}^{d};{P_{m,k}}^{d}\left(0\right),\alpha_{m,k},\beta_{m,k}\right)=\frac{\alpha_{m,k}}{\ln2}\left[\ln h_{m}+{{\tilde{P}}_{m,k}}^{d}-\ln\left(B_{1}e^{2{{\tilde{P}}_{m,k}}^{d}}+B_{2}e^{{{\tilde{P}}_{m,k}}^{d}}+N_{0}\right)\right]+\beta_{m,k}$$

Note that $\tilde{R}\left({{\tilde{P}}_{m,k}}^{d};{P_{m,k}}^{d}\left(0\right),\alpha_{m,k},\beta_{m,k}\right)$ is a concave function since it is the sum of linear and concave function terms [38]. After variable substitution, formula (17) can be rewritten as

(19) $$\tilde{\Omega }=\left\{{{\tilde{P}}_{m,k}}^{d}\;|e^{\frac{A_{{}_{2}}}{e^{{{\tilde{P}}_{m,k}}^{d}}}}+A_{2}A_{3}e^{-2{{\tilde{P}}_{m,k}}^{d}}\leq A_{3}\right\}.$$

Obviously, the function $f\left({{\tilde{P}}_{m,k}}^{d}\right)=e^{\frac{A_{{}_{2}}}{e^{{{\tilde{P}}_{m,k}}^{d}}}}+A_{2}A_{3}e^{-2{{\tilde{P}}_{m,k}}^{d}}$ is a convex function [38].

To this point, P3 can be converted into a new optimization problem as follows:

(20) $$\begin{array}{cc} P4:\\ \; & \begin{array}{c} \begin{array}{c} \max_{{{\tilde{P}}_{m,k}}^{d}\in \tilde{\Omega }}\sum_{m=1}^{M}\sum_{k=1}^{K}[\frac{\alpha_{m,k}}{\ln2}[\ln h_{m}+{{\tilde{P}}_{m,k}}^{d}-\ln(B_{1}e^{2{{\tilde{P}}_{m,k}}^{d}}+B_{2}e^{{{\tilde{P}}_{m,k}}^{d}}+N_{0})]+\beta_{m,k}]-U_{ee}\left(\sum_{m=1}^{M}\sum_{k=1}^{K}e^{{{\tilde{P}}_{m,k}}^{d}}+\sum_{m=1}^{M}P_{cir}\right) \end{array} \end{array}\\ s.t.\;\;\;\; & C1:\sum_{k=1}^{K}e^{{{\tilde{P}}_{m,k}}^{d}}\leq {P_{d}}^{\max},\;\forall m\in D\\ \; & C2:-{{\tilde{P}}_{m,k}}^{d}\leq -\ln\left(\frac{-SINR_{0}N_{0}}{K_{m}\log\left(1-p_{0}\right)}\right),\;\forall m\in D,k\in C \end{array}$$

It is easy to verify that the objective function in P4 is a concave function, the C1 in P4 is a convex function, the C2 is a affine function [38], thus P4 is a convex optimization problem according to the rule of composition and can be solved by employing the KKT conditions with the procedure illustrated in the Appendix B.

### 4.2. Channel Allocation

In this part, we aim to match DUEs for each CUE with the optimization problem as follows:

(21) $$\begin{array}{cc} P5:\;\;\;\;\;\;\;\; & \max_{\lambda_{m,k}}E\left[\frac{\sum_{m=1}^{M}\sum_{k=1}^{K}\lambda_{m,k}\log_{2}\left(1+\frac{P^{*}{{}_{m,k}}^{d}h_{m}}{A_{1}A_{3}P^{*}{{}_{m,k}}^{d}e^{\frac{-A_{2}}{P^{*}{{}_{m,k}}^{d}}}-A_{1}P^{*}{{}_{m,k}}^{d}+N_{0}}\right)}{(\sum_{m=1}^{M}\sum_{k=1}^{K}\lambda_{m,k}P^{*}{{}_{m,k}}^{d}+\sum_{m=1}^{M}P_{cir})}\right]\\ \; & s.t.\;\;\;C1:\sum_{m=1}^{M}\lambda_{m,k}\leq 1,\;\forall k\in C, \end{array}$$

where $P^{*}{{}_{m,k}}^{d}$ is the optimal power solution for PC sub-problem. To further guarantee the QoS of CUEs, we maintain a trade-off between maximizing the EE of D2D communication and minimizing the interference experienced by the BS. Then the optimal solution for CA sub-problem can be obtained as follows:

(22) $$\lambda_{m,k}=\left\{\begin{array}{cc} 1, & \;m=\underset{1\leq m\leq M}{\arg\max}\;W_{m\times k},\;\forall k\in C;\\ 0, & \;otherwise, \end{array}\right.$$

where the $W_{m\times k}$ is a M-by-K weighted utility matrix and can be defined as follows:

(23) $$W_{m\times k}=\left[\begin{array}{ccc} w_{11} & \ldots & w_{1M}\\ \vdots & \ddots & \vdots \\ w_{M1} & \ldots & w_{MK} \end{array}\right]$$

in which

(24) $$w_{mk}=\bar{w}{w_{m,k}}^{H}+\bar{\bar{w}}\left(1-{w_{m,k}}^{I}\right)$$

where $\bar{w}$,$\bar{\bar{w}}$ is the trade-off coefficient of ergodic EE and interference, respectively. ${w_{m,k}}^{H}$ is the normalized ergodic EE and ${w_{m,k}}^{I}$ is the normalized interference which can be explained respectively as follows:

(25) $${w_{m,k}}^{H}=\frac{H_{m,k}}{\sum_{m=1}^{M}H_{m,k}},$$

in which

(26) $$H_{m,k}=E\left[\frac{\log_{2}\left(1+\frac{P^{*}{{}_{m,k}}^{d}h_{m}}{P^{*}{{}_{k}}^{c}h_{k,m}+N_{0}}\right)}{P^{*}{{}_{m,k}}^{d}+P_{cir}}\right],$$

where $P^{*}{{}_{k}}^{c}$ can be derived by exploiting the relationship between ${P_{k}}^{c}$ and ${P_{m,k}}^{d}$. The $H_{m,k}$ is obtained in the following Lemma 3.

**Lemma** **3.** *The ergodic EE of the m-th DUE which reuse the channel of k-th CUE is obtained by*

(27) $$H_{m,k}=\frac{Q_{1}}{\ln(Q_{1}+Q_{2})}\left[e^{\frac{1}{Q_{1}}}E_{1}\left(\frac{1}{Q_{1}}\right)-e^{\frac{1}{Q_{2}}}E_{1}\left(\frac{1}{Q_{2}}\right)\right],$$

*where*$Q_{1}=\frac{{P_{m,k}}^{d}K_{m}}{N_{0}}$, $Q_{2}=\frac{{P_{k}}^{c}K_{k,m}}{N_{0}}$, $E_{1}\left(x\right)=\int_{0}^{x}\frac{e^{-t}}{t}dt$*is the exponential integral function of the first order.*

**Proof** **of** **Lemma** **3.** See Appendix C.

(28) $${w_{m,k}}^{I}=\frac{I_{m,k}}{\sum_{m=1}^{M}I_{m,k}}$$

where $I_{m,k}={P_{m,k}}^{d}*h_{m,k}$ indicates the interference received at the BS from the transmitter of the *m*-th pair which transmitting on the *k*-th RB. Note that the value of $w_{mk}$, $\bar{w}$, $\bar{\bar{w}}$, ${w_{m,k}}^{H}$, ${w_{m,k}}^{I}$ satisfy $w_{mk}$, $\bar{w}$, $\bar{\bar{w}}$, ${w_{m,k}}^{H}$, ${w_{m,k}}^{I}\in \left[0,1\right]$ and $\bar{w}+\bar{\bar{w}}=1$.

The proposed maximum weighted ergodic energy efficiency (MWEEE) algorithm can be described clearly in Algorithm 1. In the Algorithm 1, the proposed algorithm includes two nested algorithms. Lines 1~20 present the power control algorithm and lines 21~28 demonstrate the channel allocation algorithm. For the first-stage PC algorithm, the outer while loop execute the Dinkelbach iteration process which is proved to be three loops in the simulation results. In the inner while loop, $N_{loop}$ is the required number of iterations by the KKT solution process to obtain the optimal power value. Thus, the computational complexity is $O(3MN_{loop})$. For the second-stage CA algorithm, the computational complexity is O(MK). Thus the complexity of the whole algorithm is $Max(O\left(3MN_{loop}\right),O\left(MK\right))$. Compared with exhaustive searching based methods with the computational complexity $O(M^{K})$, the proposed scheme can considerably reduce the computational complexity.
**Algorithm 1.** Maximum Weighted Ergodic Energy Efficiency (MWEEE) Algorithm**Input:**${P_{d}}^{max};p_{0};K_{m},K_{k,m},\forall m,k;SINR_{0}$**Output:**$P^{*}{{}_{m,k}}^{d},P^{*}{{}_{k}}^{c},{\lambda^{*}}_{m,k},\forall m,k;C_{ee}^{*}$1: Initialize: the number of iteration i,j=0, the $U_{ee}\left(i\right)=0$, the threshold $\epsilon =e^{-5}$, $P_{m,k}^{d}\left(0\right)$
(∀m,k), the approximation coefficient α(j),β(j), maximum number of iterations $j^{max}$, the learning rate s1,s2, the lagrange multiplier $\mu_{1}=0.1,\mu_{2}=0.1$.2: Obtain ${P_{m,k}}^{d}$ with the given initialized parameters according to P5.3: $\Delta \leftarrow \varphi (U_{ee})$4: $P_{tmp}\leftarrow {P_{m,k}}^{d}\left(0\right)+\epsilon$5: **while**
$\varphi (U_{ee})\geq \epsilon$ (*i*-th iteration) **do**6:    **while**
$\|{P_{m,k}}^{d}\left(0\right)-P_{tmp}\|\geq \epsilon$ (*j*-th iteration) **do**7:        $P_{tmp}\leftarrow {P_{m,k}}^{d}\left(0\right)$8:        $\mu_{1}\left(j+1\right)={\left[\mu_{1}\left(j\right)-s1*\left(\sum e^{{{\tilde{P}}_{m,k}}^{d}}-{P_{d}}^{max}\right)\right]}^{+}$9:        $\mu_{2}\left(j+1\right)={\left[\mu_{2}\left(j\right)-s2*\left(-{{\tilde{P}}_{m,k}}^{d}-\ln\left(\frac{-SINR_{0}N_{0}}{K_{m}\log\left(1-p_{0}\right)}\right)\right)\right]}^{+}$10.        ${P_{m,k}}^{d}\left(0\right)\leftarrow {P_{m,k}}^{d}\left(j\right)$, obtain $ż{P_{m,k}}^{d}\left(j+1\right)$ with the ${P_{m,k}}^{d}\left(0\right)$ and then obtain α(j+1),β(j+1)11:        j←j+1
12:    **end while**13:    ${P_{m,k}}^{d}\left(i\right)\leftarrow {P_{m,k}}^{d}\left(j-1\right)$14:    Obtain $U_{ee}\left(i+1\right)$ according to the formula (4).15:    Obtain ${P_{m,k}}^{d}\left(i+1\right)$ according to P5.16:    $\Delta \leftarrow \|\varphi (U_{ee})\|$17:    i←i+118: **end while**19: Output ${P_{m,k}}^{d}\left(i\right)$ as $P^{*}{{}_{m,k}}^{d}$.20: Compute the $P^{*}{{}_{k}}^{c}$ with the $P^{*}{{}_{m,k}}^{d}$, ∀m,k.21: **for**
k=1 to *K*
**do**22:    **for**
m=1 to *M*
**do**23:         Substitute $\left(P^{*}{{}_{k}}^{c},P^{*}{{}_{m,k}}^{d}\right)$ into formula (26) and formula (28) to obtain ${w_{m,k}}^{H}$ and ${w_{m,k}}^{I}$ respectively.24:    **end for**25:    Obtain ${\lambda^{*}}_{m,k}$ according to formula (22).26: **end for**27: Compute $C_{ee}^{*}$ according to formula (8).28: Output ${\lambda^{*}}_{m,k},C_{ee}^{*}$, ∀m,k. □

## 5. Simulation Results

In this section, we present the simulation results of the proposed algorithm which is referred to as the "MWEEE" scheme to prove its effectiveness comparing with three other resource management schemes which are listed below.

1. 2-D Search: This scheme, proposed in [20], allow each DUE to reuse only one CUE and each RB not to be reused by more than one DUE. It searches for the optimal power solution of CUEs and DUEs in the region where the power value of CUE and DUE range from zero to the maximum power value. In this simulation, the subcarriers are assigned by finding the most suitable DUE for each RB to maximize the EE of DUE.
2. Restricted 2-D Search: This scheme, proposed in [21], discusses the cases of feasible regions depending on magnitudes of the maximum power of CUE and DUE. The power allocation problem is solved by using the bisection search method according to the power feasible regions. The authors assume that DUEs and CUEs is matched with one-to-one strategy. Thus, for the fairness of comparison, the subcarriers are assigned by finding the optimal RB which maximize the EE of DUE.
3. Cooperative Power: This scheme, proposed in [24], focuses on maximizing the EE of DUEs. It describes the linear power relationship between DUE and the corresponding CUE and substituted it into the optimization problem which is proved to be a convex problem and can be solved by the KKT condition. In the spectrum allocation phase, the subcarriers are assigned to the DUE which has the greatest contribution to maximizing the EE of D2D links.

**Figure 2:**
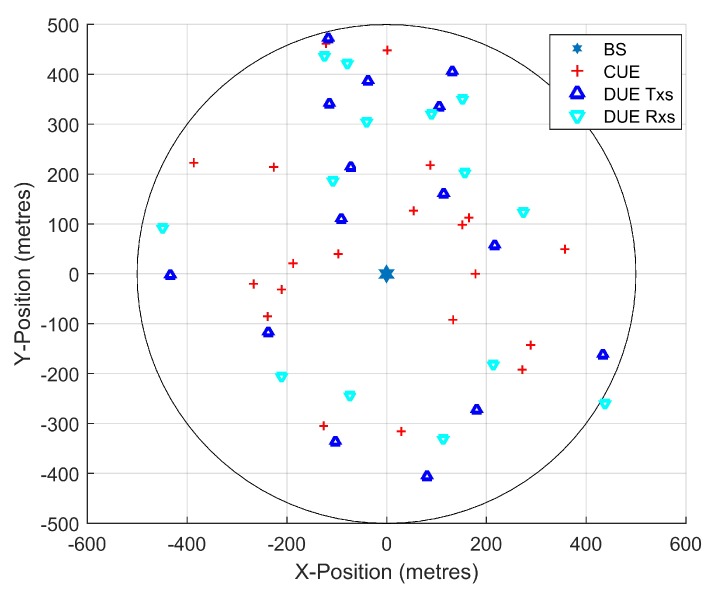
Snapshot for cellular user equipment (CUEs), D2D pairs and base station (BS) distribution (*M* = 15, *K* = 20).

We implement the simulation on the MATLAB platform and a snapshot for a single cell with a radius of 500 m is shown in Figure 2. The CUEs and DUEs distribute randomly in the cell. Each CUE is subject to interference from Gaussian white noise and a DUE that share RB with CUE and this kind of mutual interference is reflected at the BS. The receiver of D2D pair suffers from Gaussian white noise and different independent interference from CUE. The related simulation system parameter settings are given in Table 1.

**Figure 3:**
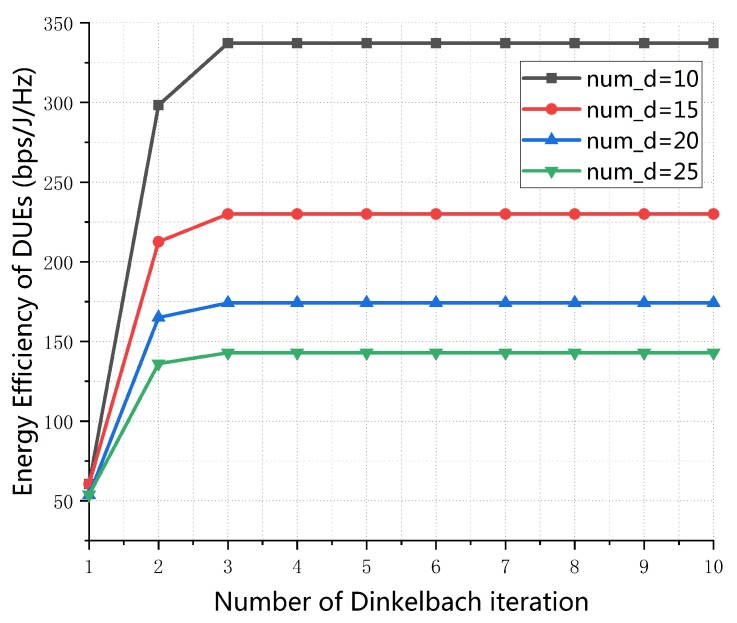
Energy-efficiency of D2D pairs vs. number of Dinkelbach iteration (K = 30, dmax = 100 m, $p_{0}$ = 0.01, $SINR_{0}$ = 20 dB).

Figure 3 plots the EE performance of D2D communications versus the number of Dinkelbach iteration of the proposed algorithm with different number of D2D pairs. The simulation result shows that it only take 3 iterations for EE of D2D communications to converge to the unique optimum value. In addition, the change of the number of D2D pairs has no effect on the convergence performance of system network, which proves the stability of the proposed algorithm.

**Figure 4:**
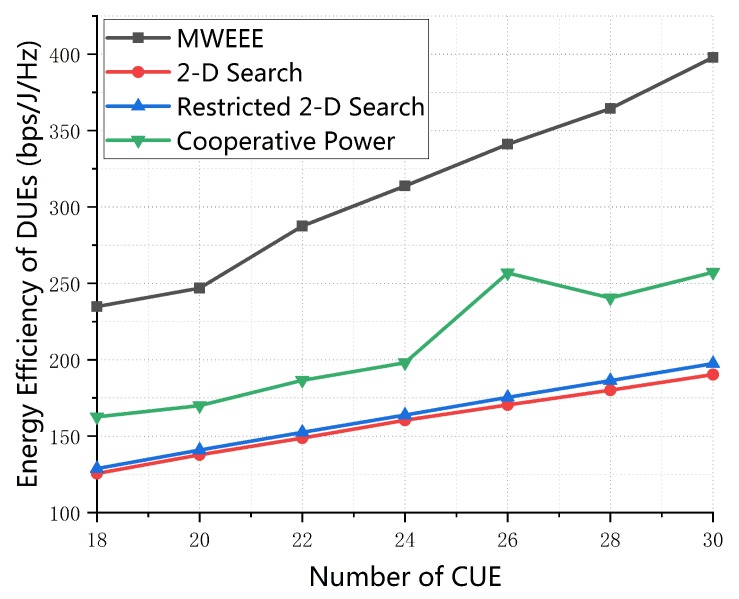
Energy-efficiency of D2D pairs vs. number of cellular user equipment (CUE) (*M* = 20, dmax = 100 m, $p_{0}$ = 0.01, $SINR_{0}$ = 20 dB).

Figure 4 compares the EE of D2D links of the proposed scheme with three other schemes. It presents that the EE of D2D links of four algorithms increase as the number of CUE increase. This is because, if there are more CUEs, the D2D pairs can communicate over more channel resources and thus the EE of D2D communications is improved. Obviously, our proposed algorithm performs much better than three other algorithms because the proposed algorithm it better at coordinating the transmit power of both CUEs and DUEs than Cooperative Power algorithm in [24] which assumed that the transmit power of CUEs varies linearly with that of DUEs, 2-D Search algorithm in [20] which implemented power control for DUEs and CUEs in the maximum power range, and Restricted 2-D Search algorithm in [21] which searched for the optimal power solution of DUEs based on whether the transmit power of CUEs is larger than the maximum transmit power of CUEs. Furthermore, the 2-D Search is close to the Restricted 2-D Search schemes because with the increase of the number of CUE, they ignored the case that there is a Collaborative relationship between CUEs and DUEs so that they controlled power for DUEs and CUEs independently. The cooperative Power algorithm in [24] shows better performance than the 2-D Search and Restricted 2-D Search. The reason for this is that the Cooperative Power scheme considers the cooperative power control between DUEs and CUEs to some extent.

**Figure 5:**
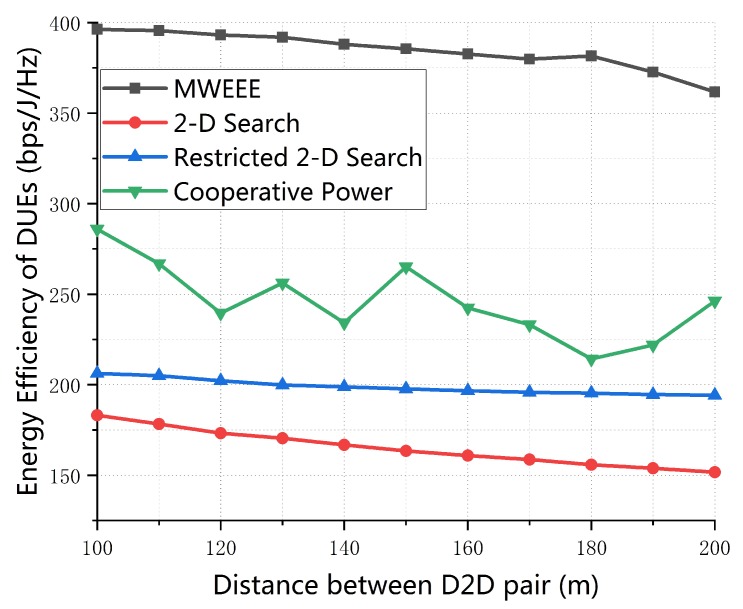
Energy-efficiency of D2D pairs vs. maximum distance of D2D communication (*M* = 20, *K* = 30, $p_{0}$ = 0.01, $SINR_{0}$ = 20 dB).

Figure 5 shows the EE performance of D2D DUEs versus the maximum transmission distance of D2D pairs referred as dmax. It can be seen from Figure 5 that the EE of D2D links decreases with the dmax. This is because the higher transmission power is required for D2D pairs to communicate normally and more path loss happens when the maximum transmission distance of D2D communication increases. It is worth mentioning that the performance of our algorithm is significantly better than the performance of the three other algorithms. The reason for this is that the proposed algorithm considered the fact that users may be mobile in the actual environment and thus cause the variety of CSI and the users power scheduling. Our MWEEE scheme reveals the effect on EE of DUEs from the actual transmit power collaboration between CUEs and DUEs. In addition, we can find that the EE of D2D links of Restricted 2-D Search algorithm in [21] is higher than that of 2-D Search scheme in [20] and the EE of D2D links of our proposed algorithm is higher than that of Cooperative Power scheme in [24]. This means that small-scale channel fading plays a part in the EE performance of D2D communication system. The results also reveal that it is inapplicable that accurate knowledge of instantaneous CSI for all links is assumed to be perfectly known at BS, especially in dense networks. Besides, the EE of D2D links of Restricted 2-D Search algorithm falls more slowly than that of the 2-D Search scheme in [20], the reason for this is that with the the communication distance changing, the Restricted 2-D Search algorithm in [21] can better adapt to the change. From the perspective of transmit power relationship between CUEs and DUEs, the performance of our proposed algorithm and Cooperative Power algorithm are both better than that of 2-D Search algorithm and Restricted 2-D Search algorithm because they control the power for DUEs while considering the power of CUEs.

**Figure 6:**
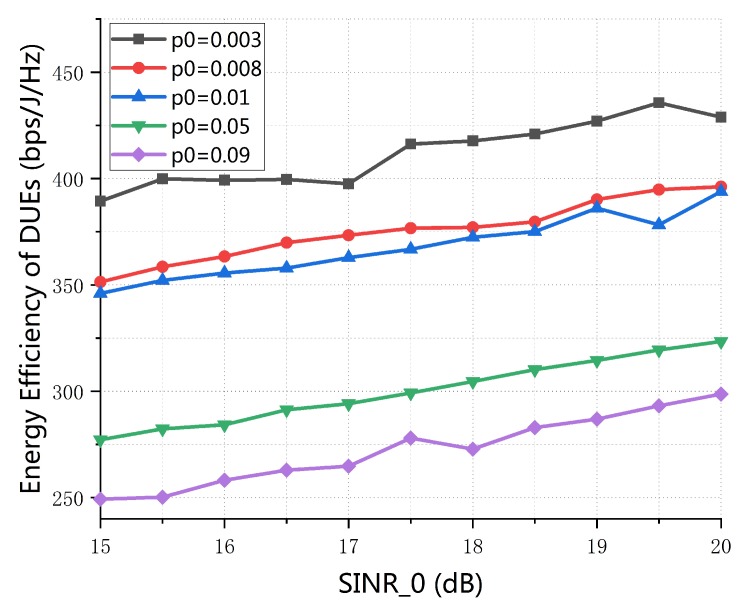
Energy-efficiency of D2D pairs vs.minimum requirement signal-to-interference-plus-noise (SINR) of D2D link (*M* = 20, *K* = 30, dmax = 150 m).

Figure 6 illustrates the EE of DUEs with the variation of minimum requirement SINR of D2D link. From Figure 6, we can see that the EE of DUEs is increasing as the $SINR_{0}$ increase. This is because from C1 in P1, with the increase of $SINR_{0}$, the SINR of DUE will increase to maximally satisfy the transmission reliability requirement of DUE. Thus the EE of DUEs increases with the $SINR_{0}$. In addition, given a fixed $SINR_{0}$ value, the EE of DUEs increase with the decrease of of value of outage probability. The reason is that with the decrease of value of outage probability, the $SINR_{0}$ increases to reduce the probability of communication interruption, which consistent with the changes in the value of $SINR_{0}$.

**Figure 7:**
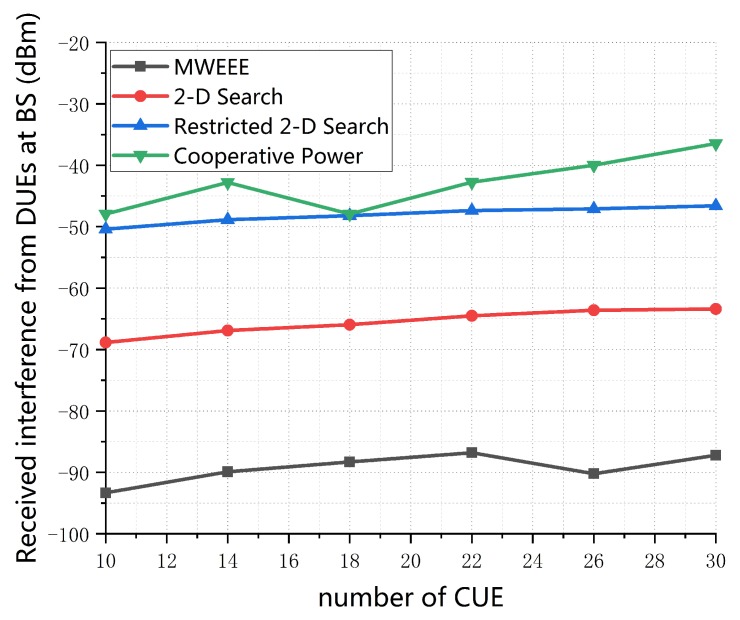
Received interference from DUEs at BS vs. number of CUEs (*M* = 20, dmax = 150 m, $p_{0}$ = 0.01, $SINR_{0}$ = 20 dB).

Figure 7 shows the received interference from DUEs at BS versus the number of CUEs. It is noted that the received interference from DUEs at BS increases with the number of CUEs on the whole. The reason for this is that a large number of CUEs provides a higher probability for the DUEs to be matched and thus causes greater interference to BS. In addition, it is apparent that the interference caused in the proposed scheme MWEEE is much less than that of three other schemes. This is because, in the Spectrum allocation stage, the MWEEE scheme maximizes the EE of DUEs while considering the interference from DUEs by introducing weight coefficient to maintain the trade-off between maximizing the EE of DUEs and minimizing the interference caused by DUEs. In the 2-D Search scheme, Restricted 2-D Search and Cooperative Power scheme, the subcarriers are assigned by finding the optimal RB which maximizes the individual EE of DUE, while ignoring the interference from DUE to BS.

## 6. Conclusions

In this paper, we have investigated the joint power control and channel allocation scheme for D2D communications underlaying cellular networks with the target of maximizing the ergodic energy efficiency of the D2D pairs. The maximum weighted ergodic energy efficiency algorithm (MWEEE) is proposed as the mechanism to control power in the PC sub-problem and to allocate RBs in the CA sub-problem. In particular, we revealed the real collaborative relationship of the transmit power between DUE and CUE when considering the statistical channel information and outage probability of the DUE. In addition, we introduced trade-off between ergodic EE of D2D communication and interference from DUEs received at the BS to further guarantee the QoS of the CUEs. In addition, simulation results demonstrate the superiority of our proposed algorithm in terms of achieving better EE performance of DUEs and less received interference from D2D to BS. As future work, we will consider the multiple cells which might utilize the full benefits of D2D communication technology. 

## Figures and Tables

**Table 1:** Simulation Parameter Settings.

Parameters	Values
Cell radius R	500 (m)
UEs distribution	randomly distributed
Number of D2D pairs	10–30
Number of cellular link	10–30
Maximum Tx power of CUE ${\left(P_{k}^{c}\right)}^{max}$	27 (dBm)
Maximum Tx power of DUE ${\left({P_{m,k}}^{d}\right)}^{max}$	25 (dBm)
uplink bandwidth B	160 (kHz)
Maximum distance between D2D-Tx and D2D-Rx	100–200 (m)
Thermal noise power	−174 (dBm)
Circuit power consumption	50 (mW)
Path loss exponent	3
Path constant	$10^{-2}$
Shadowing distribution	Log-normal
Shadowing standard deviation ζ	8 (dB)
Minimum requirement SINR of D2D link	15–20 (dB)
Tolerable outage probability $p_{0}$	0.003–0.09

## Appendix A

The constraint 1 in P1 can be given details as follows:

(A1) $$\begin{array}{c} \mathrm{Pr}\{SIN{R_{m,k}}^{d}\leq SINR_{0}\}\\ =\int_{0}^{\infty }dg_{k,m}\int_{0}^{\frac{SINR_{0}\left(N_{0}+{P_{k}}^{c}K_{k,m}g_{k,m}\right)}{K_{k,m}{P_{m,k}}^{d}}}e^{-(g_{k,m}+g_{m})}dg_{m}\\ =1-\frac{{P_{m,k}}^{d}K_{m}e^{-\frac{SINR_{0}N_{0}}{K_{m}{P_{m,k}}^{d}}}}{{P_{m,k}}^{d}K_{m}+SINR_{0}{P_{k}}^{c}K_{k,m}}\leq p_{0} \end{array}$$

Then we can get

(A2) $${P_{k}}^{c}\leq \frac{K_{m}{P_{m,k}}^{d}}{SINR_{0}K_{k,m}}\left(\frac{e^{-\frac{SINR_{0}N_{0}}{K_{m}{P_{m,k}}^{d}}}}{1-p_{0}}-1\right)$$

From the P1, the optimization objective function decreases with the increase of ${P_{k}}^{c}$ when the ${P_{m,k}}^{d}$ is given and we allocate resource for DUEs considering the worst case where ${P_{k}}^{c}$ takes the upper bound, i.e.,

(A3) $${P_{k}}^{c}=\frac{K_{m}{P_{m,k}}^{d}}{SINR_{0}K_{k,m}}\left(\frac{e^{-\frac{SINR_{0}N_{0}}{K_{m}{P_{m,k}}^{d}}}}{1-p_{0}}-1\right)$$

At this point, we have completed the proof.

## Appendix B

The P4 is a convex optimization problem, thus the point that satisfy the KKT conditions are optimal solution for both primal problem and dual problem, i.e., there have zero duality gap. Then the Lagrangian function can be written as follows:

(A4) $$\begin{array}{c} L\left({\tilde{P}}_{m,k}^{d},\mu_{1},\mu_{2}\right)=\sum_{m=1}^{M}\sum_{k=1}^{K}[\frac{\alpha_{m,k}}{\ln2}[\ln h_{m}+{\tilde{P}}_{m,k}^{d}-\ln(B_{1}e^{2{\tilde{P}}_{m,k}^{d}}+B_{2}e^{{\tilde{P}}_{m,k}^{d}}+N_{0})]+\beta_{m,k}]\\ -U_{ee}\left(\sum_{m=1}^{M}\sum_{k=1}^{K}e^{{\tilde{P}}_{m,k}^{d}}+\sum_{m=1}^{M}P_{cir}\right)-\mu_{1}\left(\sum_{k=1}^{K}e^{{\tilde{P}}_{m,k}^{d}}-{P_{d}}^{\max}\right)-\mu_{2}\left(-{\tilde{P}}_{m,k}^{d}-\ln\left(\frac{-SINR_{0}N_{0}}{K_{m}\log\left(1-p_{0}\right)}\right)\right) \end{array}$$

where $\mu_{1},\mu_{2}\geq 0$ are the Lagrange Multiplier which correspond the DUE’s power constraint. Then the KKT conditions that optimal solution must meet are given as follows:

(A5) $$\begin{array}{c} \frac{\partial L({\tilde{P}}_{m,k}^{d},\mu_{1},\mu_{2})}{\partial {\tilde{P}}_{m,k}^{d}}=0\\ \sum_{k=1}^{K}e^{{\tilde{P}}_{m,k}^{d}}-{P_{d}}^{\max}\leq 0\\ -{\tilde{P}}_{m,k}^{d}-\ln\left(\frac{-SINR_{0}N_{0}}{K_{m}\log\left(1-p_{0}\right)}\right)\leq 0\\ \mu_{1}\left(\sum_{k=1}^{K}e^{{\tilde{P}}_{m,k}^{d}}-{P_{d}}^{\max}\right)=0\\ \mu_{2}\left(-{\tilde{P}}_{m,k}^{d}-\ln\left(\frac{-SINR_{0}N_{0}}{K_{m}\log\left(1-p_{0}\right)}\right)\right)=0 \end{array}$$

Note that

(A6) $$\frac{\partial L({\tilde{P}}_{m,k}^{d},\mu_{1},\mu_{2})}{\partial {\tilde{P}}_{m,k}^{d}}=\sum_{m=1}^{M}\sum_{k=1}^{K}\frac{\alpha_{m,k}}{\ln2}\left(1-\left(\frac{e^{{\tilde{P}}_{m,k}^{d}}\left(2B_{1}e^{{\tilde{P}}_{m,k}^{d}}+B_{2}\right)}{B_{1}e^{2{\tilde{P}}_{m,k}^{d}}+B_{2}e^{{\tilde{P}}_{m,k}^{d}}+N_{0}}\right)\right)-U_{ee}\sum_{m=1}^{M}\sum_{k=1}^{K}e^{{\tilde{P}}_{m,k}^{d}}-\mu_{1}\sum_{k=1}^{K}e^{{\tilde{P}}_{m,k}^{d}}+\mu_{2}$$

Let the formula (A6) be equal to zero and substitute $e^{{\tilde{P}}_{m,k}^{d}}={P_{m,k}}^{d}$ into it, we can obtain the corresponding result as follows:

(A7) $$a{\left({P_{m,k}}^{d}\right)}^{3}+b{\left({P_{m,k}}^{d}\right)}^{2}+c\left({P_{m,k}}^{d}\right)+d=0,$$

where

(A8) $$\begin{array}{c} a=\left(U_{ee}+\mu_{1}\right)\frac{\ln2}{\alpha_{m,k}},\\ b=(2B_{1}+B_{2}U_{ee}\frac{\ln2}{\alpha_{m,k}}+B_{2}\mu_{1}\frac{\ln2}{\alpha_{m,k}}-\mu_{2}\frac{\ln2}{\alpha_{m,k}}),\\ c=\frac{\ln2}{\alpha_{m,k}}\left(U_{ee}+\mu_{1}-\frac{\mu_{2}B_{2}}{N_{0}}\right)N_{0},\\ d=-\left(\frac{\ln2}{\alpha_{m,k}}\mu_{2}+1\right)N_{0} \end{array}$$

Let

(A9) $$\begin{array}{c} \theta =-\frac{b}{3a},\;\phi =\frac{b^{2}-3ac}{9a^{2}},\\ \eta =-2a\phi^{3},\;\upsilon =\frac{2b^{3}}{27a^{2}}-\frac{bc}{3a}+d \end{array}$$

Three cases can be obtain for the solution of formula (A7). If $\upsilon -\eta^{2}>0$ [39], there exists one real root, i.e.,

(A10) $$x_{1,1}=\theta +{\left(\frac{-\upsilon +\sqrt{\upsilon^{2}-\eta^{2}}}{2a}\right)}^{\frac{1}{3}}$$

If $\upsilon -\eta^{2}=0$, there exist two real roots, i.e.,

(A11) $$\begin{array}{c} x_{2,1}=\theta +\phi ,\;x_{2,2}=\theta -2\phi \end{array}$$

If $\upsilon -\eta^{2}<0$, there exist three real roots, i.e.,

(A12) $$\begin{array}{c} x_{3,1}=\theta +2\phi \cos\left(\xi \right),\\ x_{3,2}=\theta +2\phi \cos\left(\frac{2\pi }{3}-\xi \right),\\ x_{3,3}=\theta +2\phi \cos\left(\frac{2\pi }{3}+\xi \right), \end{array}$$

where $\xi =\frac{1}{3}\arccos\left(\frac{\theta }{\eta }\right)$. With all the possible candidate optimal power solution for three cases, we can obtain the optimal transmit power ${{P^{*}}_{m,k}}^{d}$.

## Appendix C

(A13) $$H_{m,k}=E\left[\frac{\log_{2}\left(1+\frac{P^{*}{{}_{m,k}}^{d}h_{m}}{P^{*}{{}_{k}}^{c}h_{k,m}+N_{0}}\right)}{P^{*}{{}_{m,k}}^{d}+P_{cir}}\right]=\frac{E[\log_{2}\left(1+\frac{P^{*}{{}_{m,k}}^{d}h_{m}}{P^{*}{{}_{k}}^{c}h_{k,m}+N_{0}}\right)]}{P^{*}{{}_{m,k}}^{d}+P_{cir}}\overset{\Delta }{\;=}\frac{E[\log_{2}\left(1+\frac{Q_{1}X}{1+Q_{2}Y}\right)]}{P^{*}{{}_{m,k}}^{d}+P_{cir}},$$

where $Q_{1}=\frac{{P_{m,k}}^{d}K_{m}}{N_{0}}$,$Q_{2}=\frac{{P_{k}}^{c}K_{k,m}}{N_{0}}$, $h_{m}$ and $h_{k,m}$ are denoted by *X* and *Y*, respectively. We define *Z* as $Z=\frac{Q_{1}X}{1+Q_{2}Y}$.

The CDF of the variable *Z* can be given as follows:

(A14) $$F\left(z\right)=\mathrm{Pr}\left\{\frac{Q_{1}X}{1+Q_{2}Y}\leq z\right\}=\int_{0}^{\infty }dy\int_{0}^{\frac{z(1+Q_{2}y)}{Q_{1}}}e^{-(x+y)}dx=1-e^{\frac{z}{Q_{1}}}\frac{Q_{1}}{Q_{1}+Q_{2}z}$$

The statistical individual EE of *m*-th DUE is given as:

(A15) $$\begin{array}{c} H_{m,k}=\frac{\frac{1}{\ln2}\int_{0}^{\infty }\ln(1+z)f\left(z\right)dz}{P^{*}{{}_{m,k}}^{d}+P_{cir}}=\frac{\frac{1}{\ln2}\int_{0}^{\infty }\frac{1-F(z)}{1+z}dz}{P^{*}{{}_{m,k}}^{d}+P_{cir}}=\frac{\frac{1}{\ln2}\int_{0}^{\infty }\frac{e^{-\frac{z}{Q_{1}}}}{\left(1+z\right)\left(1+\frac{Q_{2}}{Q_{1}}z\right)}dz}{P^{*}{{}_{m,k}}^{d}+P_{cir}}=\frac{\frac{1}{\ln2}\left[\int_{0}^{\infty }\frac{e^{-\frac{z}{Q_{1}}}}{1+z}dz-\int_{0}^{\infty }\frac{e^{{}^{-\frac{z}{Q_{1}}}}}{\frac{Q_{1}}{Q_{2}}+Z}dz\right]\frac{Q_{1}}{Q_{1}-Q_{2}}}{P^{*}{{}_{m,k}}^{d}+P_{cir}}\\ =\frac{\frac{Q_{1}}{\ln2(Q_{1}-Q_{2})}\left[e^{\frac{1}{Q_{1}}}E_{1}\left(\frac{1}{Q_{1}}\right)-e^{\frac{1}{Q2}}E_{1}\left(\frac{1}{Q_{2}}\right)\right]}{P^{*}{{}_{m,k}}^{d}+P_{cir}} \end{array}$$
